# Comparison of Dietary Intake Before vs After Taxes on Sugar-Sweetened Beverages and Nonessential Energy-Dense Foods in Mexico, 2012 to 2018

**DOI:** 10.1001/jamanetworkopen.2023.25191

**Published:** 2023-07-24

**Authors:** Carolina Batis, Analí Castellanos-Gutiérrez, Tania G. Sánchez-Pimienta, Alan Reyes-García, M. Arantxa Colchero, Ana Basto-Abreu, Tonatiuh Barrientos-Gutiérrez, Juan A. Rivera

**Affiliations:** 1 Consejo Nacional de Ciencia y Tecnología—Center for Health and Nutrition Research, National Institute of Public Health, Cuernavaca, Mexico; 2 Center for Health and Nutrition Research, National Institute of Public Health, Cuernavaca, Mexico; 3 Harvard T.H. Chan School of Public Health, Harvard University, Boston, Massachusetts; 4 Center for Population Health Research, National Institute of Public Health, Cuernavaca, Mexico; 5 Center for Health Systems Research, National Institute of Public Health, Cuernavaca, Mexico

## Abstract

**Importance:**

Tax evaluations have shown effectiveness in reducing purchases of taxed items. However, few studies have looked at changes in overall dietary intake according to national surveys.

**Objective:**

To estimate the contribution of taxed and untaxed beverages and foods to dietary intake over time, and the nutritional dietary profile in total beverages and foods consumed.

**Design, Setting, and Participants:**

This cross-sectional study used data from 3 National Health and Nutrition Surveys of a nationally representative sample of participants aged older than 1 year in Mexico from 2012 (pretax), 2016, and 2018 (posttax). Data were analyzed from September 2021 to December 2022.

**Exposure:**

Taxes on sugar-sweetened beverages and nonessential energy-dense foods implemented since 2014.

**Main Outcomes and Measures:**

The outcomes were the contribution of taxed and untaxed beverages to total beverage volume, the contribution of taxed and untaxed foods to total food energy, and the energy density and content of added sugars and saturated fats. Outcomes were assessed with 24-hour recalls (24HR) (2012 and 2016) and Food Frequency Questionnaires (FFQ) (2012, 2016, and 2018), and were adjusted by sociodemographic and macroeconomic variables.

**Results:**

A total of 17 239 participants were analyzed from 2012, 18 974 from 2016, and 30 027 from 2018; approximately 50% were men, and approximately 75% lived in urban areas. According to 24HRs, the contribution of taxed beverages to total beverage volume changed −2.3 (95% CI, −4.4 to −0.2) percentage points from 2012 to 2016, while water increased. The contribution of taxed foods to total food energy changed −3.0 (95% CI, −4.2 to −1.8) percentage points, while untaxed whole grains, processed meats, other animal sources, and sugars and desserts increased. The content in total beverages of added sugars changed −1.1 kcal/100 mL (95% CI, −2.0 to −0.2), and in total foods, the content of added sugar changed −0.6 %kcal (95% CI, −1.0 to −0.2), saturated fat changed −0.8 %kcal (95% CI, −1.1 to −0.4), and energy density changed −9.8 kcal/100 g (95% CI, −13.8 to −5.8). Main results were consistent with the FFQ (up to 2018), with some differences in subgroups and nutritional components.

**Conclusions and Relevance:**

The findings of this study on 3 cross-sectional national dietary surveys are consistent with previous evaluations; after tax implementation, there was a decrease in the contribution of taxed items. Furthermore, some unhealthy untaxed items increased, but the content of unhealthful nutritional components, particularly added sugar, in overall beverages and foods decreased.

## Key Points

**Question:**

Were taxes on nonessential energy-dense food and sugary beverages associated with decreases in their contribution to dietary intake and an improved overall nutritional dietary profile?

**Findings:**

In this cross-sectional study using data from 3 national nutrition surveys of 75 240 participants, the contribution of taxed beverages and foods decreased while untaxed water, whole grains, processed meats, other animal sources, and sugar and desserts increased. The content of unhealthful nutritional components in total beverages and total foods, particularly added sugars, decreased.

**Meaning:**

These findings suggest that contribution of taxed items decreased, and the nutritional dietary profile improved after the tax, despite some increases in the contribution of unhealthy untaxed items.

## Teaser

This cross-sectional study compares dietary intake before and after taxes on sugar-sweetened beverages (SSBs) and nonessential energy-dense foods in Mexico.

## Introduction

Taxation is an effective policy to address the health and economic burden caused by the excessive intake of unhealthy foods and beverages.^1,2,3^ Worldwide, the number of countries or local jurisdictions implementing diet-related taxes is increasing; by 2022, more than 45 countries and jurisdictions had implemented taxes on sugar-sweetened beverages (SSB).^4^ Mexico was one of the first countries to implement diet-related taxes. Since 2014, the Mexican government implemented a 1 peso-per-liter tax (approximately 10% price increase) on SSBs and an 8% ad valorem tax on nonessential energy-dense food.^5^ The SSB tax comprises all industrialized nonalcoholic beverages (including concentrates and powders) with added sugar. The food tax includes nonessential foods with an energy density of 275kcal/100 g or more, such as chips and snacks, candies and sweets, chocolate, puddings, peanut and hazelnut butter, ice cream, ice pops, and cereal-based products with added sugar.

Previous studies in Mexico found that after the tax, prices increased by the amount of the tax, particularly in urban areas.^6^ Studies evaluating up to 3 years after tax implementation reported a 7.6% decrease in SSB purchases, while taxed food decreased 6.0%.^7,8,9,10,11^ Reductions were larger in lower socioeconomic status (SES) groups, in urban areas, and in households that used to have larger purchases of taxed products.^9,10^ In a cohort study, the probability of becoming a nonconsumer of SSBs increased by 5%.^12^ As in Mexico, studies from other countries concur that taxes are effective to achieve a reduction in the purchase of taxed products. A meta-analysis including 16 tax policies from all over the world found an average reduction of 15% in sales of SSBs after implementation of taxes.^4^

Most tax evaluation studies, including the ones from Mexico, have used purchase or sales data.^4^ Commercial consumer panels (eg, Nielsen or Kantar) have important advantages in evaluating beverage and food taxes because household purchases are collected longitudinally, frequently (ie, biweekly), and in great detail (ie, at the brand level).^13^ Thus, evaluations with consumer panels are more solid and can give more conclusive results than other data sources. However, an important limitation of these data is that information is aggregated at the household level so changes by individual characteristics such as age or sex cannot be identified. In addition, purchase data do not capture total dietary intake; hence, it misses the ability to evaluate total intake.

Dietary surveys conducted with 24-hour dietary recalls (24HRs) or Food Frequency Questionnaires (FFQ) can fill purchase data gaps by capturing total intake and at the individual level. Few previous efforts to monitor taxes have been conducted with a representative dietary survey.^14,15^ Yet, a challenge with dietary surveys is their large degree of measurement error, limiting the ability to capture absolute intake and attribute changes to small taxes.^16^ An additional weakness is that the periodicity of data collection is not frequent, and in the case of Mexico, surveys have been conducted in different seasons. Despite this, looking at the relative contribution of taxed items over time in dietary surveys is helpful to gain new insights. Our aim was to estimate the adjusted contribution of taxed and untaxed beverages and foods, and the nutritional dietary profile (added sugars, saturated fats, and energy density) in the National Health and Nutrition Surveys (ENSANUT for its acronym in Spanish) of 2012 (pretax), 2016, and 2018 (posttax).

## Methods

### Study Population

We analyzed dietary information from probabilistic and cross-sectional surveys representative of the Mexican population. ENSANUT 2012 was conducted between October 2011 and May 2012; ENSANUT 2016 from May to October 2016; and ENSANUT 2018 from July 2018 to February 2019. Informed consent was obtained from participants aged 18 years or older and from the parent or guardian of participants aged younger than 18 years. We adhered to the Strengthening the Reporting of Observational Studies in Epidemiology (STROBE) reporting guideline for cross-sectional studies where applicable. The survey protocol for each ENSANUT was approved by the ethics committee of the National Institute of Public Health. The description of the methods has been published elsewhere.^17,18,19^ For this analysis, we excluded children aged less than 1 year, breastfed children aged less than 1 year, and pregnant or lactating women. Additionally, we excluded individuals with extreme energy intakes according to the survey’s established protocols (see eFigure 1 and eFigure2 in Supplement 1).^20,21,22^

### Dietary Data

Dietary data from the ENSANUT 2012 and 2016 were collected using 2 instruments in different subsamples, a 24HR and a semiquantitative FFQ; in 2018, only FFQ was collected. The sample size for each instrument as well the collection or not of 24HR depended on the budget available. The subsamples for each 24HR or FFQ are nationally representative. Both 24HR and FFQ were collected in person by trained interviewers. The 24HR used the multiple-pass method.^20^ Interviewers recorded the types and amounts of all food items consumed in the previous day. The FFQ inquires about the frequency, portion size, and the number of portions consumed of 140 food items during the previous 7 days.^21^

Data from 24HR and FFQ were categorized into comparable food groups. For foods, complex mixed dishes were disaggregated into individual ingredients, and beverages were kept as preparations. Following tax law definitions, items were grouped into 4 main groups: taxed beverages, untaxed beverages, taxed foods, and untaxed foods. Additional subgroups were also analyzed. Energy and nutrient intakes were estimated in all surveys with the most recent food-composition tables compiled by the National Institute of Public Health.^23^ Added sugars were estimated as described previously by Sánchez-Pimienta et al.^24^ Energy density was estimated by dividing the energy content of foods consumed by their weight (kcal/100 g). To estimate the weight, water content in mixed dishes (eg, soups, rice, pasta) was imputed according to a database of standard recipes.

### Covariates

Sociodemographic and macroeconomic changes can be associated with dietary behaviors; thus, we accounted for these. We included sex, age, area of residence (urban [≥2500 inhabitants] or rural [<2500 inhabitants]), and region (North, Center, and South). With an index derived from a principal component analysis of household characteristics and goods, participants were classified into tertiles of socioeconomic status (SES) in 2012. The same SES cut points were applied in 2016 and 2018 to capture changes over time. Education level for participants aged 18 years and older was defined based on the head of the family. We included 2 macroeconomic variables at the state level: unemployment rate (percentage of unemployed population aged ≥15 years), and the log of gross domestic product (GDP per capita at 2013 prices).^25^

### Statistical Analysis

We estimated proportions or means for sociodemographic and macroeconomic variables and evaluated their changes over time with a χ^2^ or *t *test accordingly. To address the lack of comparable temporality between surveys, which primarily affects the intake of beverage during warmer months, we analyzed foods and beverages separately and focused on the percentage contribution instead of absolute intake. We estimated the contribution of taxed and untaxed beverages to the total volume of beverages (% vol), and the contribution of taxed and untaxed foods to total energy from foods (% kcal). The rationale for looking at volume instead of energy was that some beverages, such as water, are noncaloric and could not be captured in the % kcal. This analysis was conducted among all and by subpopulation groups (age, sex, urban or rural area, and SES).

We estimated the content of added sugar in all beverages (kcal/100 mL) and all foods (% kcal from foods) consumed, the content of saturated fat (% kcal from foods), and energy density (kcal/100 g from foods) in all foods consumed. Then we split the nutritional content and computed the share of taxed and untaxed items (ie, kcal of added sugar consumed in taxed beverages per 100 mL of all beverages consumed).

To estimate changes across surveys, we fitted linear regression models for each food group or nutritional component with the survey year as the variable and adjusting for all covariates. We present the estimated values from the adjusted models in 2012 and the difference (95% CI) between 2012 and the later surveys. The significance level was set at 2-tailed .05 for all statistical tests. We used Stata software, version 14.0 (StataCorp), and the survey module (svy) to consider the weighting and the complex survey design of ENSANUT. Data were analyzed from September 2021 to December 2022.

## Results

In total, 17 239 participants were analyzed from 2012, 18 974 from 2016, and 30 027 from 2018. The main characteristics of the study population are shown in Table 1. Approximately half of the sample were women, and about 1 quarter lived in rural areas. In 2012, the FFQ sample had a mean (SE) age of 30.9 (0.46) years. In 2012 the 24-hour sample had a mean (SE) age of 31.15 (0.35) years. The proportion of participants in the higher SES category or with higher education level increased over time.

**Table 1.** Sociodemographic Characteristics of Participants With Dietary Information in the Mexican National Health and Nutrition Surveys, 2012 to 2018

Characteristic	24-hr Recall			FFQ				
	No. (%)		*P* value 2012 vs 2016	No. (%)			*P* value 2012 vs 2016	*P* value 2012 vs 2018
	2012 (n = 10 096 )	2016 (n = 4134)		2012 (n = 7143)	2016 (n = 14 840 )	2018 (n = 30 027 )		
Sex								
Male	4899 (49.5)	1870 (48.7)	.68	3343 (49.2)	6277 (49.3)	14 377 (46.3)	.97	.009
Female	5197 (50.5)	2264 (51.3)		3800 (50.8)	8563 (50.7)	15 650 (53.7)		
Age group								
Children (1-4 y)	2113 (7.6)	502 (8.0)	.18	1210 (7.7)	1590 (8.4)	3067 (6.6)	.07	<.001
School-aged children (5-11 y)	2753 (16.1)	1095 (15.1)		1322 (14.6)	3092 (15.5)	5990 (12.8)		
Adolescents (12-19 y)	2056 (14.5)	1241 (17.4)		1993 (18.8)	2400 (16.4)	5181 (14.9)		
Adults (20-39 y)	1188 (27.3)	505 (25.7)		1130 (26.5)	3023 (28.4)	6091 (25.4)		
Adults (40-59 y)	969 (22.7)	499 (23.5)		962 (20.5)	2842 (20.2)	5854 (24.7)		
Adults (≥60 y)	1017 (11.8)	292 (10.2)		526 (11.8)	1893 (11.1)	3844 (15.5)		
Area								
Urban	6312 (73.0)	1948 (74.6)	.42	4606 (74.9)	7131 (74.2)	19 575 (77.1)	.69	.10
Rural	3784 (27.0)	2186 (25.4)		2537 (25.1)	7709 (25.8)	10 452 (22.9)		
Region								
North	2402 (19.8)	901 (19.5)	.91	1677 (19.9)	3225 (19.5)	6497 (20.4)	.95	.90
Center	4186 (48.6)	1751 (49.7)		2981 (48.9)	6526 (49.6)	12 213 (48.3)		
South	3508 (31.6)	1482 (30.8)		2485 (31.2)	5089 (30.9)	11 317 (31.3)		
Socioeconomic status								
Low	3679 (30.4)	1429 (21.6)	<.001	2568 (28.1)	5106 (22.4)	8022 (21.5)	<.001	<.001
Medium	3544 (32.0)	1491 (30.3)		2395 (32.2)	5988 (38.5)	9937 (30.4)		
High	2873 (37.6)	1214 (48.2)		2180 (39.7)	3746 (39.1)	12 068 (48.1)		
Education level^a^								
Preschool or less	943 (8.7)	344 (6.7)	<.001	589 (8.1)	1477 (6.9)	1917 (6.0)	<.001	<.001
Elementary	4089 (36.9)	1490 (28.2)		2904 (37.8)	5294 (29.9)	9067 (27.8)		
Secondary	2761 (26.1)	1378 (31.4)		1967 (27.0)	4768 (30.2)	9348 (28.5)		
High school	1513 (18.0)	684 (20.1)		1126 (16.8)	2349 (20.1)	5993 (23.1)		
Bachelor/professional or higher	760 (10.2)	238 (13.7)		557 (10.3)	952 (12.9)	3702 (14.7)		
State-level GDP, mean (SE), MXN/per capita	130 328 (1575)	138 963 (4940)	.11	129 566 (1316)	138 055 (3655)	140 745 (1114)	.004	<.001
State-level unemployment, mean (SE), %	4.8 (0.02)	4.0 (0.05)	<.001	4.9 (0.03)	4.0 (0.05)	3.4 (0.01)	<.001	<.001

Abbreviations: FFQ, Food Frequency Questionnaire; GDP, gross domestic product; MXN, Mexican pesos.

^a^ In the case of participants aged 19 years or younger, education level is from the head of household.

Table 2 shows the changes in taxed and untaxed beverages and food groups. According to the 24HR, the contribution of taxed beverages to total beverage volume decreased 2.3 (95% CI, −4.4 to −0.2) percentage points. Within taxed beverages, carbonated beverages did not change, but noncarbonated beverages decreased 2.1 percentage points. For untaxed beverages, homemade SSBs and juices remained unchanged, unsweetened beverages decreased 4.5 percentage points, and water increased 6.7 percentage points. According to the FFQ, the trends were similar, except for a decrease in carbonated beverages and an increase in homemade SSBs and juices which were statistically significant.

**Table 2.** Contribution to Volume of Taxed and Untaxed Beverages and Contribution to Energy of Taxed and Untaxed Foods in 2012 (Pretax), and Change to 2016 and 2018 (Posttax)^a^

Beverage or food	24-hr Recall		FFQ		
	2012, Mean (SE)	2016 vs 2012, β (95% CI)	2012, Mean (SE)	2016 vs 2012, β (95% CI)	2018 vs 2012, β (95% CI)
Total energy, kcal	1922.8 (18.3)	−73.7 (−137.7 to −9.8)	1800.2 (14.4)	153.7 (112.6 to 194.8)	−22.3 (−59.1 to 14.6)
Beverages					
All beverages, mL	1423.2 (17.6)	132.4 (58.0 to 206.7)	1580.0 (18.8)	577.3 (518.4 to 636.2)	304.4 (254.4 to 354.5)
Taxed beverages, % mL					
Overall	21.1 (0.5)	−2.3 (−4.4 to −0.2)	18.3 (0.4)	−3.2 (−4.2 to −2.2)	−3.7 (−4.8 to −2.7)
Carbonated beverages	13.7 (0.4)	−0.2 (−2.1 to 1.7)	13.6 (0.4)	−2.5 (−3.4 to −1.6)	−2.9 (−3.8 to −2.0)
Noncarbonated beverages	7.5 (0.3)	−2.1 (−3.1 to −1.1)	4.6 (0.2)	−0.7 (−1.2 to −0.2)	−0.8 (−1.3 to −0.4)
Untaxed beverages^b^					
Overall	78.6 (0.5)	2.6 (0.5 to 4.7)	81.7 (0.4)	3.2 (2.2 to 4.2)	3.7 (2.7to 4.8)
Homemade SSB and juices^c^	23.5 (0.5)	0.8 (−1.9 to 3.5)	12.0 (0.4)	5.9 (4.8 to 6.9)	7.1 (6.1 to 8.2)
Unsweetened beverages^d^	16.0 (0.4)	−4.5 (−5.8 to 3.6)	19.1 (0.4)	−5.0 (−5.9 to −4.0)	−6.1 (−7.1 to −5.1)
Water	37.3 (0.6)	6.7 (4.0 to 9.3)	48.6 (0.6)	2.4 (0.9 to 3.8)	2.9 (1.4 to 4.4)
Foods					
All foods, kcal	1571.4 (15.7)	−61.2 (−116.6 to −5.8)	1441.3 (11.9)	94.7 (61.1 to 128.3)	−14.3 (−44.6 to 15.9)
Taxed foods, % kcal					
Overall	17.5 (0.3)	−3.0 (−4.2 to −1.8)	18.6 (0.3v	−3.9 (−4.6 to −3.2)	−3.6 (−4.2 to −3.0)
Sweet bread from bakery	6.6 (0.3)	0.1 (−1.1 to 1.4)	6.1 (0.2)	−1.3 (−1.8 to −0.8)	−0.7 (−1.2 to −0.8)
Packaged sweet bread and cookies^e^	4.8 (0.2)	−1.5 (−2.1 to −0.9)	5.3 (0.2)	−1.6 (−2.0 to −1.2)	−2.7 (−3.0 to −2.3)
Other taxed foods^f^	6.1 (0.2)	−1.6 (−2.2 to 1.1)	7.2 (0.1)	−1.0 (−1.3 to −0.6)	−0.2 (−0.5 to 0.1)
Untaxed foods, % kcal					
Overall	82.4 (0.3)	3.1 (1.9 to 4.3)	81.4 (0.3)	3.8 (3.1 to 4.5)	3.6 (2.9 to 4.2)
Whole grains	22.6 (0.4)	1.6 (0.1 to 3.0)	27.1 (0.4)	3.8 (3.3 to 4.3)	2.1 (1.7 to 2.5)
Refined grains and tubers	15.2 (0.3)	0.9 (−0.4 to 2.2)	11.7 (0.2)	−1.6 (−1.8 to −1.3)	−1.5 (−1.7 to −1.2)
Legumes and nuts	4.7 (0.2)	−0.2 (−0.8 to 0.3)	4.5 (0.1)	−0.9 (−1.1 to −0.6)	−1.4 (−1.6 to −1.1)
Fruits	3.8 (0.2)	0.3 (−0.3 to 0.9)	6.8 (0.1)	0.5 (0.1 to 0.9)	−0.2 (−0.6 to 0.2)
Vegetables	3.0 (0.1)	−0.1 (−0.4 to 0.1)	4.5 (0.1)	0.8 (0.5 to 1.1)	0.9 (0.6 to 1.1)
Processed meats	1.7 (0.1)	0.5 (0.1 to 0.9)	1.6 (0.0)	0.1 (0.0 to 0.2)	0.1 (0.0 to 0.1)
Other animal sources^g^	19.2 (0.3)	1.6 (0.3 to 3.4)	18.5 (0.2)	1.2 (0.6 to 1.7)	2.4 (1.9 to 2.9)
Fats	10.5 (0.2)	−0.6 (−1.5 to 0.3)	4.5 (0.1)	−0.1 (−0.4 to 0.2)	0.9 (0.6 to 1.2)
Sugars and desserts	1.1 (0.1)	0.5 (0.1 to 0.9)	1.3 (0.0)	0.3 (0.2 to 0.5)	0.0 (−0.1 to 0.1)

Abbreviations: FFQ, Food Frequency Questionnaire; SSB, sugar-sweetened beverage.

^a^ Adjusted by age group, sex, urban/rural area, region, socioeconomic status, education level of adults aged 20 years or older and education level of the head of the household for participants aged less than 20 years, log-transformed state-level gross domestic product, and state-level unemployment rate.

^b^ Includes alcohol and the following subgroups: homemade SSB and juices, unsweetened beverages, and water.

^c^ Includes not bottled/homemade sweetened fruit drinks, aguas frescas, coffee, tea or milk, and juices.

^d^ Includes unsweetened fruit drinks, coffee, tea, milk, and industrialized beverages with artificial sweeteners.

^e^ Includes packaged sweet bread and cookies, cereal bars, and pastries.

^f^ Includes salty snacks, ready-to-eat cereals, chocolate, candies, ice cream, sorbets, spreads, and jellies.

^g^ Includes poultry, eggs, unprocessed red meat, cheese, and seafood.

In the case of foods (Table 2), according to the 24HR, the energy contribution to total foods of taxed foods decreased 3.0 (95% CI, −4.2 to −1.8) percentage points. Sweet bread from bakeries did not decrease, packaged sweet bread and cookies decreased 1.5 percentage points, and other taxed foods decreased 1.6 percentage points. Results with FFQ were similar, except that sweet bread from bakeries did decrease, and other taxed foods returned to 2012 levels in 2018. According to both 24HR and FFQ, the contribution of whole grains, processed meats, other animal sources, and sugar and desserts increased. The remaining food groups did not change (24HR), refined grains and legumes and nuts decreased, and all the remaining food groups increased in at least 1 survey (FFQ).

According to the 24HR, the total beverage added sugar, food added sugar, food saturated fat, and food energy density decreased after the tax (Table 3). The content in total beverages of added sugars changed −1.1 kcal/100 mL (95% CI, −2.0 to −0.2), and in total foods, the content of added sugar changed −0.6 %kcal (95% CI, −1.0 to −0.2), saturated fat changed −0.8 %kcal (95% CI, −1.1 to −0.4), and energy density changed −9.8 kcal/100 g (95% CI, −13.8 to −5.8). In beverages, the decrease was associated with untaxed beverages, likely associated with the increase in water. In foods the decrease was associated with the taxed foods. According to the FFQ, beverage added sugar and food added sugar decreased, total food saturated fat decreased in 2016 but increased in 2018, and total food energy density decreased only in 2016.

**Table 3.** Nutritional Dietary Profile in Total Foods and Beverages Consumed in 2012 (Pretax), 2016, and 2018 (Posttax) Subdivided by Taxed and Untaxed Items^a^^,^^b^

Beverage or Food	24-hr Recall		FFQ		
	2012, mean (SE)	2016 vs 2012, β (95% CI)	2012, mean (SE)	2016 vs 2012, β (95% CI)	2018 vs 2012, β (95% CI)
Beverages					
Added sugar from beverages, kcal/100mL					
Overall	12.4 (0.2)	−1.1 (−2.0 to −0.2)	13.2 (0.4)	**-**2.8 (−3.5 to −2.0)	−2.9 (−3.8 to −2.1)
Added sugar from taxed beverages	7.2 (0.2)	−0.4 (−1.2 to 0.5)	7.9 (0.2)	−1.4 (−1.9 to −1.0)	−1.6 (−2.0 to −1.1)
Added sugar from untaxed beverages	5.2 (0.2)	−0.7 (−1.3 to −0.1)	5.3 (0.3)	−1.3 (−2.0 to −0.7)	−1.4 (−2.1 to −0.6)
Foods					
Added sugar from foods, % kcal					
Overall	5.1 (0.1)	−0.6 (−1.0 to −0.2)	7.0 (0.1)	−1.1 (−1.4 to −0.8)	−1.3 (−1.5 to −1.0)
Added sugar from taxed foods	3.6 (0.1)	−0.9 (−1.2 to −0.7)	5.2 (0.1)	−1.1 (−1.3 to −0.9)	−1.0 (−1.2 to −0.8)
Added sugar from untaxed foods	1.5 (0.1)	0.3 (−0.0 to 0.6)	1.9 (0.0)	0.0 (−0.1 to 0.2)	−0.2 (−0.3 to −0.1)
Saturated fat from foods, % kcal					
Overall	11.5 (0.1)	−0.8 (−1.1 to −0.4)	10.9 (0.1)	−0.3 (−0.5 to −0.0)	0.6 (0.4 to 0.8)
Saturated fat from taxed foods	3.0 (0.1)	−0.6 (−0.8 to −0.3)	2.8 (0.0)	−0.4 (−0.5 to −0.3)	−0.2 (−0.3 to −0.1)
Saturated fat from untaxed foods	8.5 (0.1)	−0.2 (−0.6 to 0.2)	8.1 (0.1)	0.1 (−0.1 to 0.3)	0.8 (0.6 to 1.0)
Energy density from foods, kcal/100 g					
Overall	192.6 (1.1)	−9.8 (−13.8 to −5.8)	163.1 (0.8)	−5.7 (−7.7 to −3.8)	1.0 (−0.8 to 2.8)
Energy density from taxed foods	31.5 (0.7)	−5.9 (−9.0 to −2.9)	31.9 (0.5)	−7.2 (−8.6 to −5.8)	−5.8 (−7.1 to −4.6)
Energy density from untaxed foods	159.6 (1.0)	−2.9 (−6.1 to 0.4)	131.2 (0.7)	1.5 (−0.2 to 3.2)	6.8 (5.3 to 8.4)

Abbreviation: FFQ, Food Frequency Questionnaire.

^a^ Adjusted by age group, sex, urban/rural area, region, socioeconomic status, education level of adults aged 20 years or older and education level of the head of the household for participants aged less than 20 years, log-transformed state-level gross domestic product, and state-level unemployment rate.

^b^ Subdivision by taxed/untaxed categories means that the nutritional content in total beverages or foods is divided by the parts consumed in taxed/untaxed items (eg, not the nutritional content in each taxed/untaxed item).

In the Figure we present changes in the contribution of all taxed beverages and foods (see eTable in Supplement 1 for taxed subgroups) by sociodemographic characteristics. According to 24HR, taxed beverages decreased after the tax among children aged 5 to 11 years, adolescents, women, those in urban areas, and in those with medium SES. Taxed foods decreased after the tax in all age groups, sexes, urban areas, and in those with low and medium SES. The other subpopulations did not change before and after the tax. According to the FFQ, the contribution of taxed beverages and foods decreased across all sociodemographic characteristics.

**Figure.**
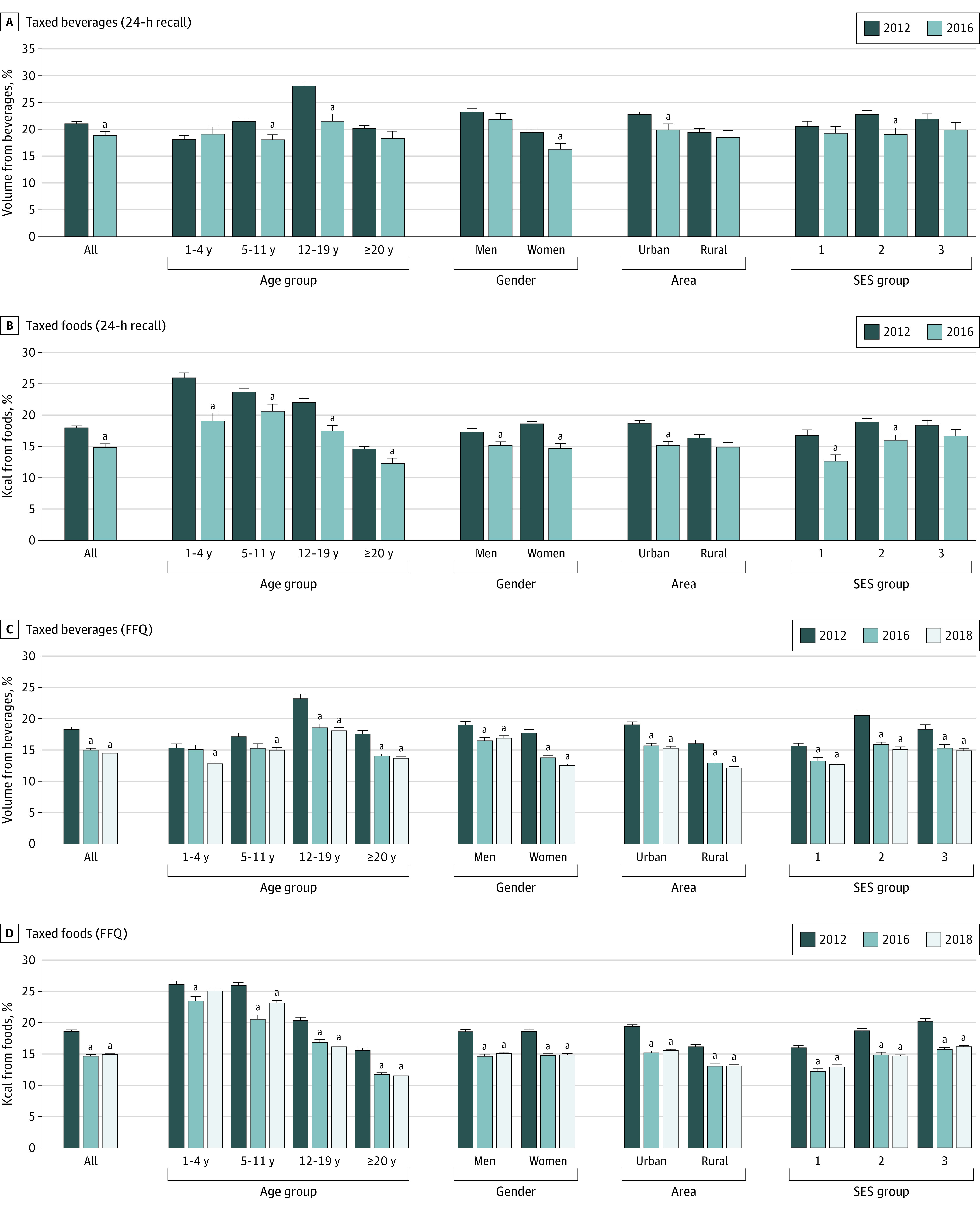
Contribution of Taxed/Untaxed Groups in 2012 (Pretax), 2016, and 2018 (Posttax) by Sociodemographic Characteristics Contribution of taxed beverages to total volume from beverages and of taxed foods to total energy from foods. Adjusted by log-transformed state-level gross domestic product, state-level unemployment rate, education level of adults aged 20 years or older and education level of the head of the household for participants aged less than 20 years, and all other sociodemographic characteristics in the figure when not stratified by it. FFQ indicates Food Frequency Questionnaire; SES, socioeconomic status. ^a^*P* < .05 vs 2012.

## Discussion

We evaluated the contribution of taxed and untaxed beverages and foods from 2012 (pretax) to 2016 and 2018 (posttax) with data from 24HR and FFQ. Despite small size taxes in Mexico and large measurement error of dietary data, we found an overall decrease in the contribution of taxed beverages to the total volume of beverages consumed, in the contribution of taxed foods to the total energy of foods consumed, and in content of unhealthful nutritional components, particularly added sugars, in total beverages and total foods.

By subpopulations and food subgroups, our findings were aligned with previous evaluations of the Mexican tax. Studies on commercial consumer panels and/or household expenditure surveys found that among those with higher SES or living in rural areas, the response was lower for SSB or null for energy-dense food^7,8,10^; or that taxed carbonated beverages had a lower reduction and taxed sweet bread from bakeries did not decrease.^7,10^ In our study, we found no change in higher SES, rural areas, carbonated beverages, and sweet bread from bakeries with the 24HR; but with the FFQ, we found statistical differences across all subpopulations and food subgroups. This suggests that the 24HR might be more precise and detailed to capture variations by subpopulations and food subgroups. Yet, in comparison with the FFQ that captures intake of last the 7 days, the 24HR has a larger random measurement error that affects statistical significance. Worldwide, the lower change among those with higher SES has been inconsistent^3,26^; for instance in Chile^27^ and the UK,^28^ the opposite was found. The finding of a larger decrease in noncarbonated vs carbonated was also observed in Chile,^27^ but the opposite was found in Barbados^29^ and Seattle.^30^ The lower change in rural areas observed in Mexico could be associated with the industry strategy of not completely passing the tax to consumers in rural areas.^31^ Finally, the finding that sweet bread from bakeries (eg, unpackaged) did not decrease could be due to a tax exemption given to small bakeries.

An important contribution of our study was the possibility to look at changes by age group and sex. According to the 24HR, taxed beverages did not change among children aged less than 5 years, adults, and men, but according to the FFQ, taxed beverages decreased in all age groups and sexes. It is likely that adults and men were less responsive because proportionally they consume fewer noncarbonated beverages (see eTable in Supplement 1), the beverages that decreased the most after the tax. Moreover, for both taxed beverages and taxed foods, we found that the age group with the largest intake before the tax had the largest decline after the tax (adolescents for beverages and children aged <5 years for foods). This is consistent with previous evaluations of the taxes in Mexico in which households with the highest baseline intake of taxed items were the ones that decreased the most after the tax.^9,32^

Another contribution was the ability of our study to look at changes in a wider range of untaxed categories. For instance, previous studies found an increase in bottled water,^7^ yet it was not possible to identify if this was used to prepare homemade sugary drinks. Homemade aguas frescas are heavily consumed in Mexico as it is unusual to have main meals with plain water. We found that along with an increase in the contribution of water to total beverage volume, there was also an increase in homemade sugary drinks (only statistically significant with FFQ). Yet, the overall content of added sugar in beverages decreased. In the case of untaxed foods, we found that food subgroups that increased their contribution according to both 24HR and FFQ were whole grains, processed meats, other animal sources, and sugars and desserts. Processed meats and sugars and desserts are unhealthy foods that are potentially substitutes of taxed energy-dense foods. Despite this, the overall intake of added sugars from foods decreased throughout, and the overall content of saturated fat and energy density from foods decreased in both 24HR and FFQ, but only up to 2016. With the FFQ in 2018, saturated fat increased and energy density returned to pretax levels. Likely, the latter was associated with an increase in other animal sources and fats. The increase in other animal sources observed with both 24HR and FFQ might be associated with an ongoing upward trend, particularly in poultry and eggs,^33^ which are affordable proteins and have become cheaper over time.^34^ Changes between taxed and untaxed subgroups analyzed were limited to their contribution (ie, % vol, % kcal) and did not include changes in absolute amounts (ie, mL or kcal). Dietary surveys are prone to a large degree of measurement error, making it difficult to assess changes in energy intake in absolute terms.^16,35^ For instance, it is difficult to know how much of the 73 kcal per day that decreased from 2012 to 2016 in the 24HR is due to measurement error. In a previous analysis, we evaluated the intraindividual change among a subsample of the ENSANUT 2012 with 2 repeated 24HRs and found that energy intake was not fully substituted, as on the days in which participants did not consume taxed beverages or foods, total energy intake decreased.^36^ Thus, a decrease in total energy intake associated with the taxes is a possibility.

### Limitations and Strengths

National dietary surveys provide useful information for tax evaluation, yet they are limited in important ways. The degree of measurement error is large, as explained before. Also, before the tax, there were no repeated surveys in short and frequent intervals, so we did not account for previous ongoing trends, and there are substantial lags between our studied periods and the implementation of the tax. The months of data collection between 2012 and 2016 were not comparable, and although our analysis focused on percentage volume to address the increased intake of beverages in the warmer months, seasonality effects within beverage contributions can remain. Nonetheless, the months were comparable in approximately 82% of the sample between 2012 and 2018. Desirability bias could also influence the results, particularly if the tax raised awareness^37^ of the unhealthfulness of SSBs and energy-dense foods. Yet, the quality of dietary data collection and the frequency with which the ENSANUT is collected is remarkable, particularly for a middle-income country. The use of 2 dietary instruments across 3 surveys that are representative of the national population is a key strength of our study. Furthermore, despite the limitations, our results are consistent with previous tax evaluations in Mexico.

## Conclusions

Our analysis of national dietary nutrition surveys, despite its limitations, yielded similar conclusions to prior evaluations of taxes in Mexico using different data sources, contributing to the consistency of results. As a new contribution, we documented some increases in the contribution of unhealthy untaxed items after the tax, but these did not offset the decreases in the overall content of unhealthful nutritional components, particularly added sugars, in total beverages and foods. Our results also suggest that changes in the contribution of taxed beverages was heterogenous by age and sex. Altogether, our findings suggest that despite their small size, taxes in Mexico have contributed to improve the quality of dietary intake, and thus, these should be augmented to or beyond levels recommended by the World Health Organization. Our results reiterate that taxes remain a key part of the strategy for obesity and chronic disease prevention.

## Notes

Supplement 1. **eTable. **Contribution to Volume of Taxed and Untaxed Beverages and Contribution to Energy of Taxed and Untaxed Foods in 2012 (Pretax) and 2016 (Posttax) by Sociodemographic Characteristics According to 24-Hour Dietary Recalls **eFigure 1. **Flowchart of Final Analytical Sample With 24-hr Dietary Recalls **eFigure 2. **Flowchart of Final Analytical Sample With Food Frequency Questionnaires

Supplement 2.
Data Sharing Statement
