# Supplementary material for: Comparison of Dietary Intake Before vs After Taxes on Sugar-Sweetened Beverages and Nonessential Energy-Dense Foods in Mexico, 2012 to 2018
Source: JAMA Netw Open. 2023 Jul 24;6(7):e2325191. doi: 10.1001/jamanetworkopen.2023.25191 (PMC10366703; doi:10.1001/jamanetworkopen.2023.25191)
Supplement: Supplement 1. — eTable. Contribution to Volume of Taxed and Untaxed Beverages and Contribution to Energy of Taxed and Untaxed Foods in 2012 (Pretax) and 2016 (Posttax) by Sociodemographic Characteristics According to 24-Hour Dietary Recalls eFigure 1. Flowchart of Final Analytical Sample With 24-hr Dietary Recalls eFigure 2. Flowchart of Final Analytical Sample With Food Frequency Questionnaires [file jamanetwopen-e2325191-s001.pdf]

Supplemental Online Content

Batis C, Castellanos-Gutiérrez A, Sánchez-Pimienta TG, et al. Comparison of dietary intake before vs after taxes on sugar-sweetened beverages and nonessential energy-dense foods in Mexico, 2012 to 2018. *JAMA Netw Open*. 2023;6(7):e2325191. doi:10.1001/jamanetworkopen.2023.25191

eTable. Contribution to Volume of Taxed and Untaxed Beverages and Contribution to Energy of Taxed and Untaxed Foods in 2012 (Pretax) and 2016 (Posttax) by Sociodemographic Characteristics According to 24-Hour Dietary Recalls
eFigure 1. Flowchart of Final Analytical Sample With 24-hr Dietary Recalls
eFigure 2. Flowchart of Final Analytical Sample With Food Frequency Questionnaires

This supplemental material has been provided by the authors to give readers additional information about their work.

© 2023 Batis C et al. *JAMA Network Open.*

eTable. Contribution to Volume of Taxed and Untaxed Beverages and Contribution to Energy of Taxed and Untaxed Foods in 2012 (Pretax) and 2016 (Posttax) by Sociodemographic Characteristics According to 24-Hour Dietary Recalls[1]

	2012 Mean ± SE	2016 vs 2012 β (95% CI)	2012 Mean ± SE	2016 vs 2012 β (95% CI)	2012 Mean ± SE	2016 vs 2012 β (95% CI)	2012 Mean ± SE	2016 vs 2012 β (95% CI)
	**Children 1-4y**		**Children 5-11y**		**Adolescents**		**Adults**	
Sample size, n	2,113	502	2,753	1,095	2,056	1,241	3,174	21,296
Total Energy, kcal	1347.6 ± 21.7	-142.1 (-230.5, -53.8)	1818.7 ± 24.9	-146.7 (-236, -57.3)	2071 ± 30.7	-59.8 (-159.4, 39.8)	1996.8 ± 27.6	-52.9 (-150.7, 45)
All beverages, ml	897.8 ± 20.8	64.7 (-29.4, 158.8)	1111.9 ± 23.1	9.1 (-58.3, 76.4)	1425.6 ± 29.3	205.7 (114.1, 297.3)	1568.9 ± 27.1	146.9 (31.2, 262.6)
Taxed beverages, % ml from beverages	18.1 ± 0.8	1 (-1.9, 3.8)	21.4 ± 0.8	-3.3 (-5.7, -1)	28.1 ± 1	-6.6 (-10.1, -3)	20.1 ± 0.7	-1.7 (-4.8, 1.3)
Carbonated beverages, % ml from beverages	5.8 ± 0.5	2 (-0.1, 4)	10.0 ± 0.6	1.1 (-1.1, 3.4)	18.3 ± 0.8	-3.6 (-6.4, -0.9)	14.7 ± 0.6	-0.2 (-3.1, 2.6)
Non-carbonated beverages, % ml from beverages	12.4 ± 0.7	-1.1 (-3.7, 1.5)	11.4 ± 0.6	-4.5 (-6.2, -2.7)	9.8 ± 0.8	-3 (-5, -1)	5.4 ± 0.4	-1.5 (-3, -0.1)
All foods, kcal	1019.7 ± 20.8	-94 (-175.7, -12.4)	1511.9 ± 22.2	-131.7 (-213.5, -50)	1719.7 ± 28.4	-41.4 (-138.7, 55.9)	1627.8 ± 23.6	-46.2 (-130.2, 37.7)
Taxed foods, % kcal from foods	25.3 ± 0.8	-6.7 (-9.6, -3.8)	23.1 ± 0.6	-3 (-5.4, -0.6)	21.4 ± 0.7	-4.3 (-6.6, -2)	14.2 ± 0.4	-2.2 (-4, -0.4)
Sweet bread from bakery, % kcal from foods	7.8 ± 0.6	-3.5 (-5.2, -1.9)	6.0 ± 0.4	-1.1 (-2.6, 0.5)	5.7 ± 0.5	0.3 (-1.2, 1.7)	6.7 ± 0.4	0.9 (-1, 2.8)
Packaged sweet bread and cookies[2], % kcal from foods	6.7 ± 0.5	-0.1 (-2.2, 1.9)	6.5 ± 0.4	-1.5 (-2.8, -0.2)	6.2 ± 0.4	-2.1 (-3.3, -1.9)	3.8 ± 0.3	-1.6 (-2.4, -0.7)
Other taxed foods[3], % kcal from foods	10.8 ± 0.5	-3 (-4.8, -1.1)	10.6 ± 0.4	-0.4 (-2, 1.1)	9.5 ± 0.5	-2.5 (-4.2, -2.1)	3.7 ± 0.2	-1.5 (-2.2, -0.9)
	**Men**		**Women**		**Urban**		**Rural**	
Sample size, n	4,899	1, 870	5, 197	2, 264	6, 312	1, 948	3, 784	2, 186
Total Energy, kcal	2135.7 ± 29.6	-32.7 (-132.4, 67.1)	1725.1 ± 21.1	-110.4 (-192.6, -28.2)	1945.0 ± 22.7	-92.6 (-174.2, -10.9)	1889.9 ± 31.7	-46.5 (-135.6, 42.5)
All beverages, ml	1567.0 ± 29.7	203.3 (86.4, 320.2)	1290.6 ± 20.1	68.9 (-12.5, 150.4)	1472.7 ± 22.4	114.6 (23.2, 206.1)	1344.8 ± 32.4	152.6 (43.3, 261.9)
Taxed beverages, % ml from beverages	23.2 ± 0.7	-1.4 (-4.1, 1.3)	19.4 ± 0.7	-3.1 (-5.9, -0.3)	22.7 ± 0.6	-2.8 (-5.6, 0)	19.4 ± 0.8	-0.9 (-3.5, 1.7)
Carbonated beverages, % ml from beverages	16.1 ± 0.7	-0.4 (-2.9, 2.1)	11.5 ± 0.5	0.1 (-2.4, 2.6)	14.6 ± 0.5	-0.6 (-3.1, 1.9)	12.8 ± 0.7	0.8 (-1.5, 3.1)
Non-carbonated beverages, % ml from beverages	7.1 ± 0.5	-1 (-2.6, 0.6)	7.9 ± 0.4	-3.2 (-4.3, -2.1)	8.1 ± 0.4	-2.2 (-3.6, -0.8)	6.6 ± 0.4	-1.7 (-2.6, -0.8)
All foods, kcal	1733.6 ± 24.5	-53.3 (-135.7, 29.1)	1418.2 ± 18.7	-65.8 (-142.3, 10.6)	1573.6 ± 19.5	-65.6 (-136.6, 5.5)	1568.7 ± 28.4	-60.8 (-139.8, 18.1)
Taxed foods, % kcal from foods	16.9 ± 0.5	-2.1 (-3.6, -0.6)	18.1 ± 0.4	-3.9 (-5.8, -1.9)	18.2 ± 0.4	-3.4 (-5, -1.9)	15.9 ± 0.6	-1.4 (-3, 0.3)
Sweet bread from bakery, % kcal from foods	6.5 ± 0.4	0.9 (-0.5, 2.3)	6.7 ± 0.4	-0.6 (-2.6, 1.4)	6.4 ± 0.3	0.5 (-1.1, 2.2)	6.3 ± 0.5	-0.2 (-1.6, 1.1)
Packaged sweet bread and cookies[2], % kcal from foods	5.0 ± 0.3	-1.6 (-2.5, -0.6)	4.6 ± 0.2	-1.4 (-2.2, -0.6)	5.0 ± 0.2	-1.9 (-2.6, -1.1)	4.3 ± 0.4	-0.6 (-1.8, 0.5)
Other taxed foods[3], % kcal from foods	5.4 ± 0.3	-1.4 (-2.1, -0.7)	6.7 ± 0.2	-1.9 (-2.7, -1)	6.8 ± 0.2	-2.1 (-2.8, -1.4)	5.4 ± 0.3	-0.5 (-1.1, 0.1)
	**SES1**		**SES2**		**SES3**			
Sample size, n	3,679	1, 429	3, 544	1, 491	2843	1, 214		
Total Energy, kcal	1900.2 ± 39.2	-70.4 (-162.3, 21.6)	1936.2 ± 29.2	-72.8 (-176.4, 30.8)	1908.9 ± 33.2	-81.6 (-196.6, 33.5)		
All beverages, ml	1350.9 ± 45.1	169.6 (25.4, 313.7)	1380.9 ± 31.3	159.7 (42.5, 276.8)	1455.3 ± 35.1	67.4 (-66.6, 201.5)		
Taxed beverages, % ml from beverages	20.5 ± 1	-1.3 (-3.6, 1)	22.7 ± 0.9	-3.7 (-6.6, -0.9)	21.9 ± 1.1	-2.1 (-6.3, 2.2)		
Carbonated beverages, % ml from beverages	13.1 ± 0.8	-0.1 (-2.3, 2.2)	15.2 ± 0.7	-1.3 (-4, 1.3)	13.9 ± 1	0.3 (-3.8, 4.4)		

© 2023 Batis C et al. *JAMA Network Open.*

Non-carbonated beverages, % ml from beverages	7.5 ± 0.6	-1.3 (-2.7, 0.2)	7.5 ± 0.5	-2.4 (-3.7, -1.1)	8.0 ± 0.6	-2.4 (-4.5, -0.3)
All foods, kcal	1568.1 ± 33.7	-101.3 (-174.5, -28)	1582.8 ± 24.6	-61.2 (-152.2, 29.7)	1537.1 ± 31.6	-31.4 (-147.3, 84.6)
Taxed foods, % kcal from foods	16.3 ± 0.9	-4 (-5.5, -2.4)	18.4 ± 0.6	-2.8 (-4.8, -0.8)	17.8 ± 0.7	-1.7 (-4.4, 0.9)
Sweet bread from bakery, % kcal from foods	7.5 ± 1	-2.1 (-3.3, -0.9)	7.5 ± 0.4	-0.1 (-1.6, 1.4)	5.2 ± 0.7	2.6 (-0.3, 5.5)
Packaged sweet bread and cookies[2], % kcal from foods	4.1 ± 0.4	-0.9 (-1.8, 0.1)	4.5 ± 0.3	-1.1 (-2.3, 0.1)	5.5 ± 0.4	-2.2 (-3.3, -1.1)
Other taxed foods[3], % kcal from foods	4.7 ± 0.4	-1 (-1.6, -0.4)	6.4 ± 0.3	-1.6 (-2.6, -0.6)	7.1 ± 0.4	-2.1 (-3.2, -1.1)

[1]Adjusted by log-transformed state-level GDP, state-level unemployment rate, education level of adults aged ≥20y and education level of the head of the household for participants under 20y of age, and all other sociodemographic characteristics in the table when not stratified by it.
[2]Includes packaged sweet bread and cookies, cereal bars, and pastries
[3]Includes salty snacks, ready-to-eat cereals, chocolate, candies, ice cream, sorbets, spreads, jellies.

© 2023 Batis C et al. *JAMA Network Open.*

eFigure 1. Flowchart of Final Analytical Sample With 24-hr Dietary Recalls

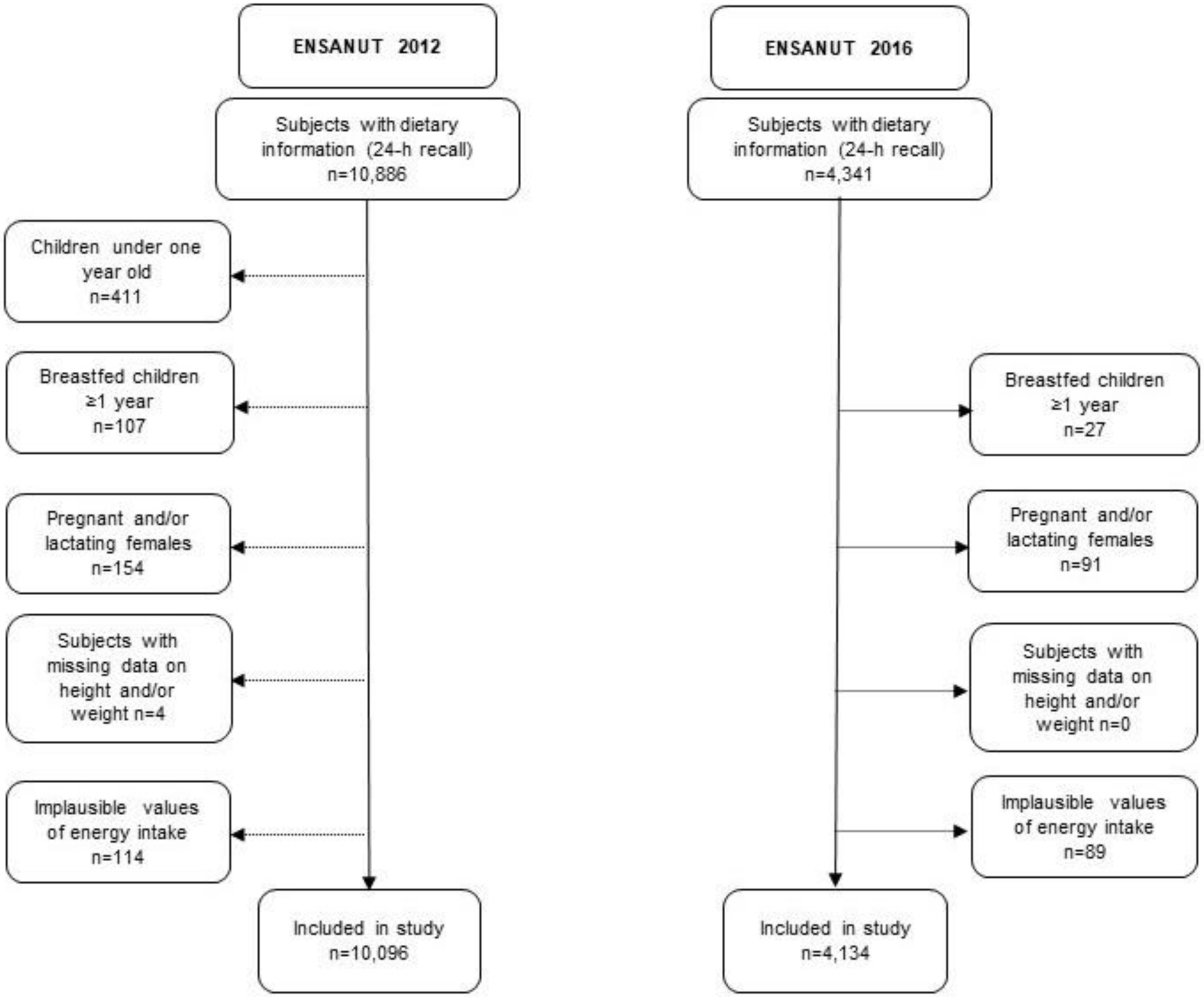

eFigure 2. Flowchart of Final Analytical Sample With Food Frequency Questionnaires

© 2023 Batis C et al. *JAMA Network Open.*

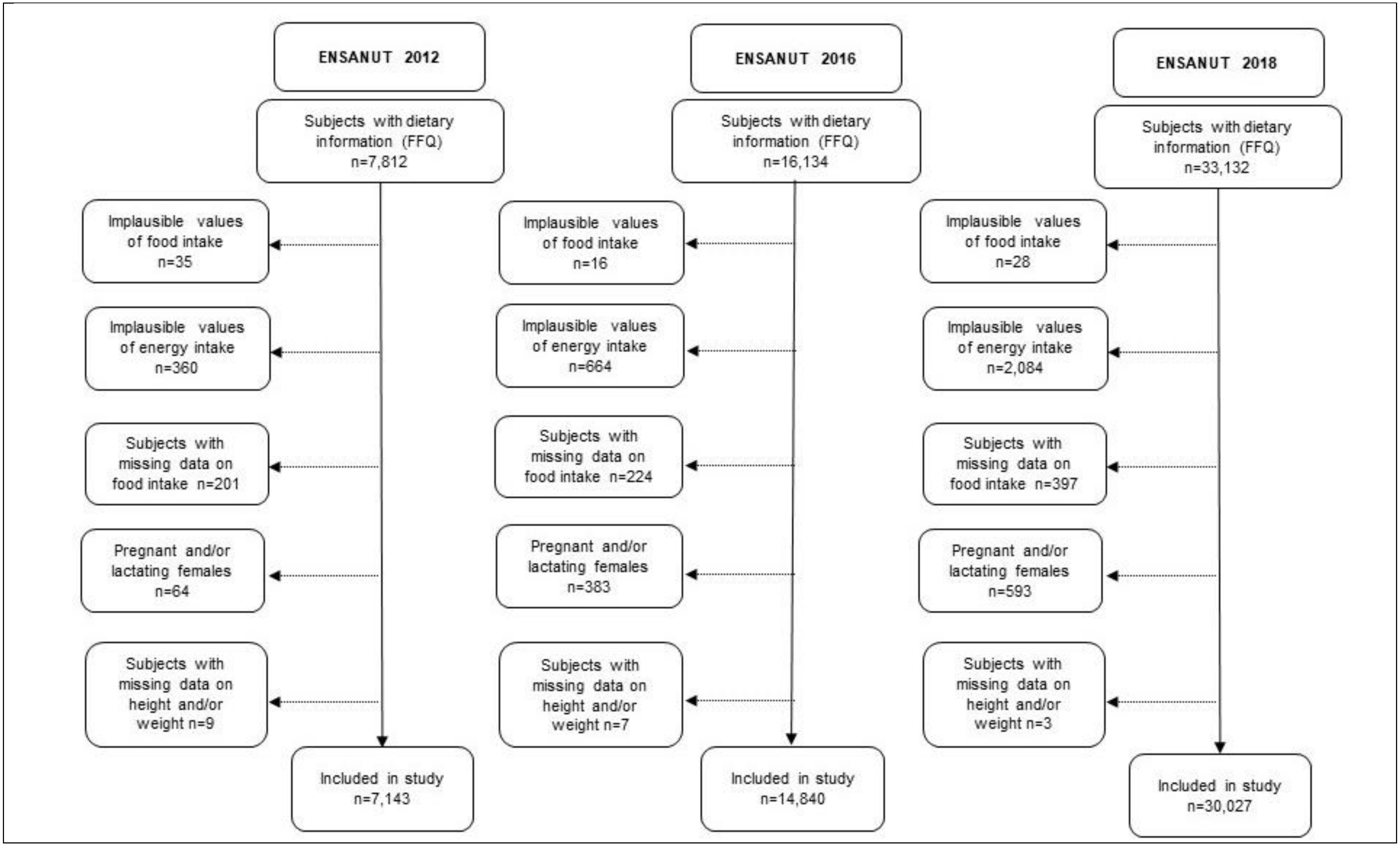

© 2023 Batis C et al. *JAMA Network Open.*
