# Supplementary material for: Comparison of Dietary Intake Before vs After Taxes on Sugar-Sweetened Beverages and Nonessential Energy-Dense Foods in Mexico, 2012 to 2018
Source: JAMA Netw Open. 2023 Jul 24;6(7):e2325191. doi: 10.1001/jamanetworkopen.2023.25191 (PMC10366703; doi:10.1001/jamanetworkopen.2023.25191)
Supplement: Supplement 2. — Data Sharing Statement [file jamanetwopen-e2325191-s002.pdf]

# Data Sharing Statement

Batis. Comparison of Dietary Intake Before vs After Taxes on Sugar-Sweetened Beverages and Nonessential Energy-Dense Foods in Mexico, 2012 to 2018. *JAMA Netw Open*. Published July 24, 2023. doi:10.1001/jamanetworkopen.2023.25191

**Data**
**Data available:** Yes
**Data types:** Deidentified participant data, Data dictionary
**How to access data:** https://ensanut.insp.mx/
**When available:** beginning date: 11-01-2020

**Supporting Documents**
**Document types:** None

**Additional Information**
**Who can access the data:** data is publicly available
**Types of analyses:** data is publicly available for any purpose
**Mechanisms of data availability:** data is publicly available without any requirement
